# Validating the Existential Quest Scale using item response theory

**DOI:** 10.3389/fpsyg.2025.1716603

**Published:** 2026-01-09

**Authors:** Marco Rizzo, Giorgia Molinengo, Barbara Loera, Anna Miglietta, Vassilis Saroglou

**Affiliations:** 1 Department of Theoretical and Applied Sciences, e-Campus University, Novedrate, Italy; 2 Department of Psychology, University of Turin, Turin, Italy; 3 Psychological Sciences Research Institute, Université catholique de Louvain, Louvain-la-Neuve, Belgium

**Keywords:** differential item functioning, existential quest, psychometric properties, Rasch analysis, uncertainty

## Abstract

The Existential Quest Scale (EQS) is a brief instrument designed to assess individuals’ willingness to engage with existential quest in both religious and secular contexts. As the construct of existential quest becomes increasingly relevant for understanding psychological flexibility, identity development, and social attitudes in multicultural societies, ensuring the validity of its measurement is essential. Previous validations of the EQS have relied on Classical Test Theory (CTT), which limits comparability across groups and item-level precision. This study aims to evaluate the EQS using Rasch modeling, a robust item response theory (IRT) approach that overcomes such limitations. Drawing on a large, heterogeneous sample (*N* = 4,378), we assessed dimensionality, item functioning, and measurement invariance across sex, age, and religious affiliation. Results confirmed the unidimensional structure of the EQS, its ability to discriminate levels of existential quest, and its psychometric invariance across demographic groups. Findings also suggested revisions to item and response category functioning to enhance scale performance. By applying IRT to the EQS, this study advances the psychometric assessment of complex, culturally adaptive constructs and supports the EQS as a rigorous tool for research and applied settings.

## 1 Introduction

In recent years, researchers have emphasized the importance of existential quest for psychological wellbeing, resilience, and personal growth (Deci and Ryan, 2012). The human capacity to reflect on question such as the inevitability of death, personal freedom, and one’s place in the worlds shapes how individuals navigate adversity and cultural complexity across lifespan (Arrowood et al., 2022), including when these reflections trigger distress when accompanied by uncertainty (Weems et al., 2004).

Traditionally, existential meaning-making has been studied within religious or spiritual spheres (Steger et al., 2006). The concept of Religious Quest (RQ), for instance, refers to an individual’s active engagement with existential issues through the lens of faith, emphasizing openness, doubt, and the pursuit of religious understanding (Batson et al., 1993). However, this approach applies primarily to religious individuals (Arrowood et al., 2022) and may not adequately capture existential quest in increasingly secular and diverse societies.

In recent years, researchers have turned their attention to how nonreligious individuals address existential issues. With the decline of religion in modern societies and in an era of growing social complexity and cultural pluralism, Van Pachterbeke et al., (2012) introduced the concept of Existential Quest (EQ), which extends beyond the religious framework. Indeed, EQ captures an individual’s willingness to engage with existential questions from both religious and nonreligious perspectives, emphasizing uncertainty, the positive value of doubt, and openness to change viewpoints over time.

Numerous studies have explored how individuals engage with existential beliefs, thoughts, and anxiety (Allan and Shearer, 2012; Steger et al., 2006; Weems et al., 2004), but none of these constructs are directly comparable to EQ, which uniquely captures an individual’s willingness and capacity for flexibility in addressing existential questions.

Although EQ is a relatively new construct, it has already been demonstrated that it can be important not only for promoting greater wellbeing (Joshanloo, 2020), but also for defining the person in complex situations and even for the ability to manage interpersonal relationships in an increasingly complex world (Rizzo et al., 2022). Over time, EQ has been applied in various research contexts across multiple countries, consistently demonstrating its validity, broad applicability and cultural invariance (Saroglou et al., 2020).

Furthermore, Deak and Saroglou (2015, 2017) examined how EQ shapes people’s reasoning in moral dilemmas such as abortion, child euthanasia, gay adoption, and suicide, showing that individuals with higher EQ tend to engage more deeply with the existential dimensions of these issues. This individual capacity to reflect on existential matters may also be valuable in complex circumstances beyond moral decision-making—for example, in the acculturation process of second-generation Muslim immigrants in Europe, where it can help individuals manage the pressures of living between two cultures and religions (Rizzo et al., 2022). Extending this perspective to the domain of social attitudes, Uzarevic et al., (2021) showed that higher levels of EQ can reduce atheists’ prejudice toward religious people, while Miglietta et al., (2023) found that engaging in existential quest can reduce prejudice toward minorities more generally. This evidence highlights the broad practical significance of EQ as a construct, beyond its philosophical foundations.

Despite its theoretical importance, the Existential Quest Scale (EQS)—the main tool used to measure EQ—has been developed and validated primarily within the framework of Classical Test Theory (CTT) (Rizzo et al., 2019; Saroglou, 2025). While useful, CTT suffers from known limitations, such as sample dependence of reliability coefficients, item difficulty, and item discrimination (Finbråten et al., 2019). Rasch modeling offers a more rigorous framework for evaluating the EQS than Classical Test Theory (CTT), which has characterized previous validations. While CTT provides useful information at the scale level, it cannot verify item-level invariance, category functioning, or whether observed raw scores form a linear continuum. Conversely, Rasch analysis enables the assessment of scale dimensionality, the hierarchical arrangement of items, and the consistency of item difficulties across demographic subgroups. These properties make Rasch modeling particularly suitable for assessing a construct such as EQ, which is conceptualized as a general disposition and is expected to operate similarly across religious and non-religious respondents.

This study presents the first Rasch-based evaluation of the EQS, providing item-level evidence of construct validity, measurement invariance, and the functioning of its response categories.

## 2 Materials and methods

### 2.1 Participants and procedures

The present study is based on a secondary analysis of multiple independent datasets in which the EQS was previously administered. We retrieved the original data from six empirical studies, all collected using a snowball sampling procedure, that employed the EQS within broader investigations of existential meaning, religion, identity, and social attitudes (Deak and Saroglou, 2015, 2017; Miglietta et al., 2023; Rizzo et al., 2022; Saroglou et al., 2020; Uzarevic et al., 2021).

The initial sample included 6,398 participants. To harmonize these sources into a single unified dataset, we conducted a multi-step data integration procedure based on four criteria.

The first criterion was the presence of a nine-item version of the EQS in each dataset, which led to the exclusion of two datasets and a total of 439 participants from studies that used an eight-item EQS version (Deak and Saroglou, 2015, 2017).

The second criterion was the exclusion of cases with missing values through listwise deletion. A total of 152 participants were excluded from three datasets (*N* = 4, Rizzo et al., 2022; *N* = 139, Saroglou et al., 2020; *N* = 9, Uzarevic et al., 2021).

The third criterion related to the aim of testing measurement invariance across sex, age, and religious affiliation. One dataset was excluded because religious information was missing (*N* = 1,138, Miglietta et al., 2023).

The fourth and final criterion was the exclusion of participants who did not report information on sex, age, or religious affiliation. A total of 279 participants were excluded (*N* = 220, Saroglou et al., 2020; *N* = 69, Uzarevic et al., 2021).

The final sample included 4,378 participants from three datasets (*N* = 439, Rizzo et al., 2022; *N* = 2,859, Saroglou et al., 2020; *N* = 1,080 Uzarevic et al., 2021).

Data were collected from 2010 to 2019 across 15 countries. In all original studies, data were collected through self-administered questionnaires, distributed either online (e.g., university mailing lists, social media recruitment, survey platforms, cultural and religious associations) or in paper-and-pencil format where applicable. A non-probabilistic snowball sampling procedure was used for each study and was based on voluntary participation. Each study obtained ethical approval from the respective institutional review boards, and all participants provided informed consent before taking part in the research.

Table 1 summarizes the main characteristics of the studies included in this secondary analysis. Although each dataset covered a wide age range, the mean age showed that the samples consisted mainly of young adults. All studies reported that participants were primarily students and predominantly female, except for Uzarevic et al., (2021), where gender distribution was more balanced.

**Table 1:** Summary of characteristics of datasets included in the final merged database.

Study source	Years of data collection	Sample size before cleaning	Sample size after cleaning	Countries included (%)	Gender	Religious affiliation (%)	Mean age (SD)	Range
Rizzo et al. (2022)	2019	445	439	Belgium (45.8%); Italy (54.2%).	Female (71.5%); male (28.5%)	Catholics (0.9%); Protestants (0.2%); Muslims (97.0%); Other (1.8%).	26.24 (8.38)	18–62
Saroglou et al. (2020)	2012–2016	3,218	2,859	Belgium (5.5%); Costa Rica (5.4%); France (5.0%); Germany (7.6%); Greece (5.2%); Israel (5.0%); Italy (9.2%); Poland (5.2%); Slovakia (5.2%); Spain (15.6%); Switzerland (5.1%); USA (10.9%); Turkey (7.9%); Taiwan (7.2%).	Female (71.1%); male (28.9%)	Catholic (35.8%); Protestants (7.6%); Jewish (4.6%); Muslims (8.4%); Orthodox (4.2%); Agnostics (9.8%); Atheists (19.8%); Other (9.8%).	21.86 (4.75)	18–80
Uzarevic et al. (2021)	2016	1,158	1,080	France (20.3%), Spain (31.3%); UK (48.4%).	Female (52.7%); male (47.3%)	Catholics (21.3%); Protestants (4.1%); Jewish (0.4%); Muslims (1.0%); Orthodox (8.6%); Agnostics (22.8%); Atheists (34.9%); Other (6.9%).	27.09 (9.61)	18–68

Most participants who identified as atheist or agnostic were from the studies by Uzarevic et al., (2021) and Saroglou et al., (2020), while nearly all Muslim participants were from the study by Rizzo et al., (2022). This distribution reflects the aims of the individual studies, which examined prejudice among atheists and agnostics (Uzarevic et al., 2021) and the acculturation of young Muslims (Rizzo et al., 2022).

The final large dataset consisted mainly of women (65.5%), young adults with a mean age of 23.59 years (SD = 7.08; range 18–80), and mostly religiously affiliated participants (66.5%).

**Figure 1:**
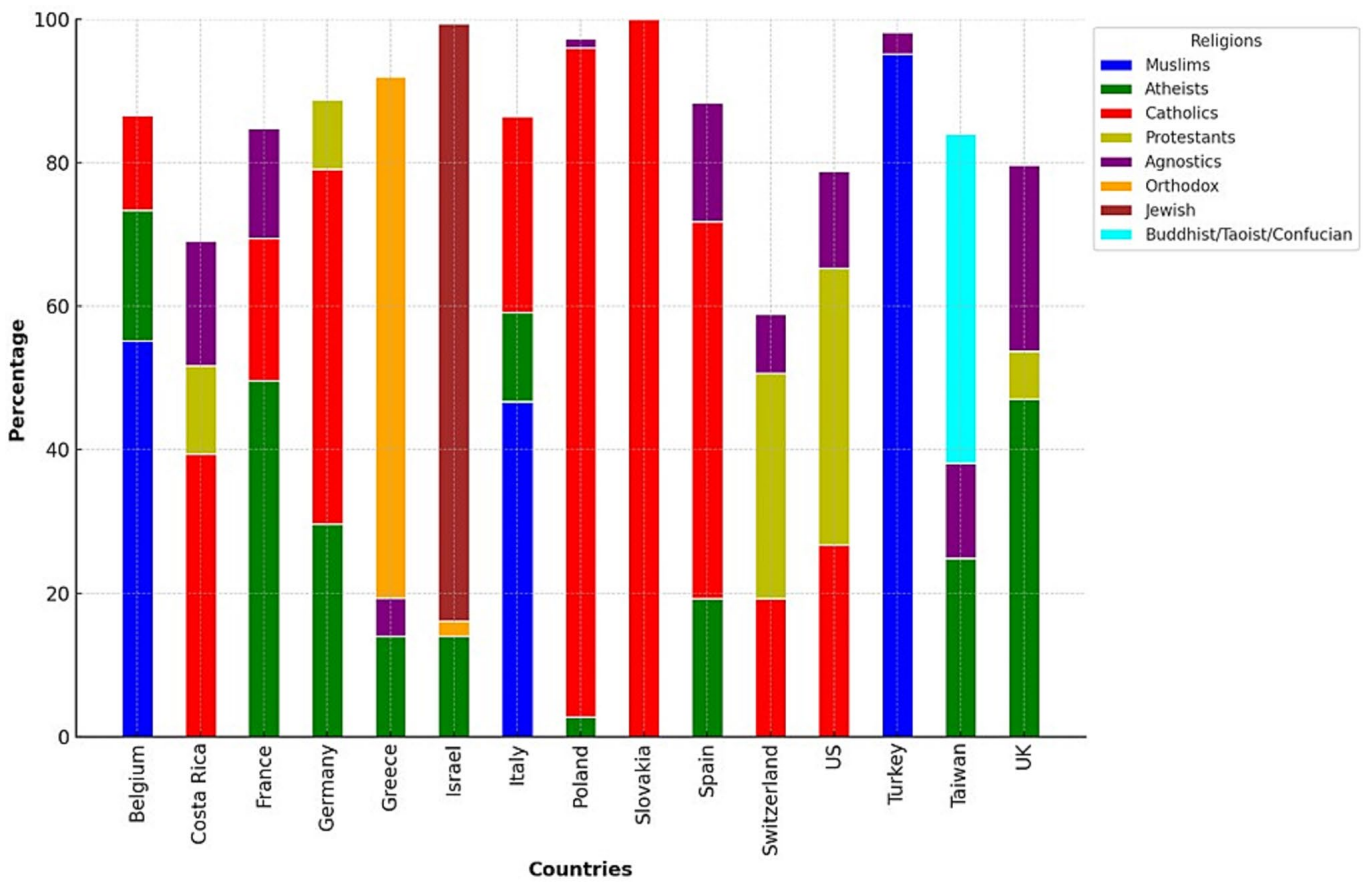
Religious confession for each country.

Figure 1 shows the distribution of countries and religious affiliation (or not).

### 2.2 Measure

The EQS measures individual flexibility in existential questions (Van Pachterbeke et al., 2012) and is composed of nine items that address uncertainty about existential issues, positive evaluation of doubt, and openness to reconsidering one’s perspective. Participants rated their responses on a 7-point Likert-type scale ranging from 1, “completely disagree” to 7, “completely agree.” High scores on the EQS indicate a high level of existential quest. Reliability was acceptable (*α* = 0.64, *ω* = 0.65) and consistent with previous studies (Van Pachterbeke et al., 2012; Rizzo et al., 2019; Saroglou, 2025). Table 2 reports conceptual domains, means, standard deviations, skewness and kurtosis of each item.

**Table 2:** Descriptive analyses.

Item	Conceptual domain	Mean	SD	S	K
EQ1: Today, I still wonder about the meaning and goal of my life	Uncertainty about existential issues	4.63	1.92	−0.42	−1.06
EQ2: My attitude toward religion/spirituality is likely to change according to my life experiences	Openness to reconsidering one’s perspective	3.75	2.02	0.09	−1.31
EQ3: Being able to doubt about one’s convictions and to reappraise them is a good quality	Positive evaluation of doubt	5.30	1.58	−0.84	0.05
EQ4: In my opinion, doubt is important in existential questions	Positive evaluation of doubt	5.11	1.61	−0.75	−0.08
EQ5: My way of seeing the world is certainly going to change again	Openness to reconsidering one’s perspective	5.10	1.68	−0.68	−0.39
EQ6: My opinion varies on a lot of subjects	Uncertainty about existential issues	4.97	1.64	−0.56	−0.50
EQ7(R): I know perfectly well what the goal of my life is	Uncertainty about existential issues	4.00	1.90	0.01	−1.15
EQ8(R): Years go by but my way of seeing the world does not change	Openness to reconsidering one’s perspective	3.70	2.04	0.17	−1.30
EQ9: I often reappraise my opinion on religious/spiritual beliefs	Openness to reconsidering one’s perspective	3.31	1.88	0.37	−1.03

S, skewness; K, kurtosis; (R), reversed item; all items ranged 1–7.

### 2.3 Statistical analysis

The data were analyzed to fully exploit the potential of the item response theory (IRT) framework, drawing on models based on the seminal work of Rasch (1960). By adopting a Rasch framework, several psychometric limitations inherent in Classical Test Theory (CTT) were thoroughly considered. CTT assumes that raw scores have a linear relationship with the latent trait and that summed scores are treated as interval-level measures. However, this assumption may be violated when response categories are ordinal, unequally spaced, or not functioning as intended. Furthermore, the CTT model operates under the assumption of independence between measurement error and true scores. Empirical evidence, however, shows that biases often occur at the extremes of the scale, compromising the equal-interval property and distorting the resulting scores (Allen and Yen, 2002; Massof, 2011). Rasch measurement theory addresses these limitations by providing a probabilistic framework in which person and item parameters are estimated independently, enabling the derivation of invariant measurements and the empirical testing of fundamental measurement criteria such as additivity, sufficiency, and specific objectivity (Wright, 1996; Combrinck, 2020). Rasch models also allow verification of linearity through separation indices (Harwell et al., 2002), evaluation of item hierarchy stability across groups, and detailed inspection of response patterns at the item and category levels. These features support the development of equal-interval, bias-reduced estimates and the assessment of scale consistency across sex, age, and religious affiliation (Bond and Fox, 2007).

The application of Rasch modeling in the present study is justified by these methodological advantages, as the dimensionality, item functioning, category structure, and measurement invariance of the EQS were evaluated using a large, heterogeneous sample.

To assess the psychometric properties of the EQS, both the Partial Credit Model (PCM; Wright and Masters, 1982) and the Rating Scale Model (RSM; Andrich, 1988) were estimated, depending on the item response structure. The PCM allows each item to have a unique rating scale structure, while the RSM assumes a common rating scale structure for all items.

We chose to adhere exclusively to Rasch-derived specifications rather than adopting more flexible models such as the two-parameter logistic (2PL) model, based on considerations of parsimony and robustness. By setting item discrimination to be identical across items (typically set to 1), Rasch models ensure that the latent trait is the sole determinant of the likelihood of endorsing an item. This constraint promotes interpretability, supports the development of invariant measurement scales, and aligns with the nature of the construct being assessed which reflects a typical rather than maximal performance trait.

Model parameters were estimated using a robust joint maximum likelihood estimation (JMLE) method. We acknowledge that alternative estimation methods, such as conditional maximum likelihood (CML) and marginal maximum likelihood (MML), are often recommended in the context of long-standing discussions of the JMLE. However, CML does not provide estimates of personal ability and relies on conditioning on individual parameters, which was incompatible with our goal of examining individual-level differences and their correlates. MML, in turn, requires the specification of a distribution for personal ability, which is problematic for typical performance constructs such as existential quest, where population distributions are unlikely to follow strong parametric shapes. Given these considerations, and the mitigating effect of our large sample size on the classic limitations of the JMLE (Chen et al., 2019), we deemed the JMLE to be the most appropriate and transparent estimator for the purposes of this study.

*Post-hoc* principal component analysis (PCA) of residuals was used to determine the scale dimension, considering a critical value of ≤ 2.0 for the eigenvalue as the rule of thumb in the identification of a one-dimensional construct.

To evaluate each item, the INFIT and OUTFIT mean square statistics were considered; their empirical values must be close to the ideal value of 1.0 or within the acceptable range of 0.5–1.5 to claim that the item fitted the model satisfactorily. The correlation between each single-item score and the Rasch measure (i.e., the Point-Measure correlation) was examined and only values ≥0.30 were considered acceptable in contributing to the scale rating. Category fit statistics (i.e., thresholds) and category probability curves were used as diagnostic tools for response-scale functioning.

The ability of the scale to differentiate participants with different levels of EQ was evaluated using the PSI-Person Separation Index (Wright and Linacre, 1994) which can be used to establish the number of statistically distinct levels [(4PSI + 1)/3] of a person’s ability.

Finally, measurement invariance across sex (1 = male; 2 = female), age (0 = 18–31 years; 1 = > 31 years, according to Rizzo et al., 2019), and religion [(with atheist and agnostic participants classified as non-religious believers (0), and all others reporting a religious affiliation as religious believers (1)] was tested by performing uniform Differential Item Functioning (DIF) analysis, using the following DIF contrast logit ranges: <0.43 = negligible, 0.43–0.64 = moderate, and >0.64 = large (Linacre, 2012). Statistical analyses were performed using IBM SPSS software v. 28 and Winsteps. For inferential tests, exact *p*-values were reported.

## 3 Results

### 3.1 Rasch analysis

Overall, the items adequately fitted both model specifications for polytomous items (PCM and RSM). Analysis of the category threshold estimates showed that the threshold structures were highly consistent across items. This provides empirical support for using the rating scale model instead of the partial credit model. The rating scale model is further justified by its greater parsimony, as it imposes a common threshold structure across all items.

Beyond the first contrast eigenvalue (1.89), the Rasch dimension accounted for 36.3% of the total variance, whereas the first residual contrast explained only 13.4%, both well within recommended thresholds. *Post-hoc* PCA of the standardized residuals further indicated that the first contrast fell below the commonly used eigenvalue cutoff of 2.0, suggesting the absence of any meaningful secondary dimension. Although items EQ7R and EQ8R showed the highest positive loadings and items EQ2–EQ5 moderate negative loadings, the magnitude of this contrast does not point to substantive multidimensionality. Together with the Rasch-based reliability coefficient of 0.97—reflecting excellent measurement precision and strong internal consistency—these indices provided robust support for the essential unidimensionality of the EQS.

Table 3 shows the items in order of misfit: all the INFIT (min = 0.73, max = 1.44) and OUTFIT (min = 0.72, max = 1.5) statistics were in the 0.5–1.5 range indicating satisfactory fit and showing that the items contributed meaningfully to the measurement of the latent construct. Consistently, most items showed a PT-measure correlation values similar and appreciable ranging from 0.35 to 0.58; only one item (EQ7) revealed a slightly lower correlation (0.29), suggesting that its contribution to the latent continuum is weaker than the other items. This may reflect a partial conceptual misalignment or local noise associated with the reverse wording, in line with the results of previous studies.

**Table 3:** EQ 9 items, misfit order: location and fit statistics (rating scale model).

Item	Location logits	INFIT MNSQ	OUTFIT MNSQ	PT-measure correlation
EQ8(R)	0.22	1.44	1.50	0.35
EQ7(R)	0.19	1.33	1.44	0.29
EQ1	−0.07	1.09	1.08	0.51
EQ9	0.50	1.01	1.02	0.51
EQ2	0.31	1.00	0.99	0.55
EQ6	−0.17	0.85	0.88	0.48
EQ3	−0.39	0.78	0.75	0.57
EQ5	−0.27	0.76	0.74	0.58
EQ4	−0.31	0.73	0.72	0.58

**Figure 2:**
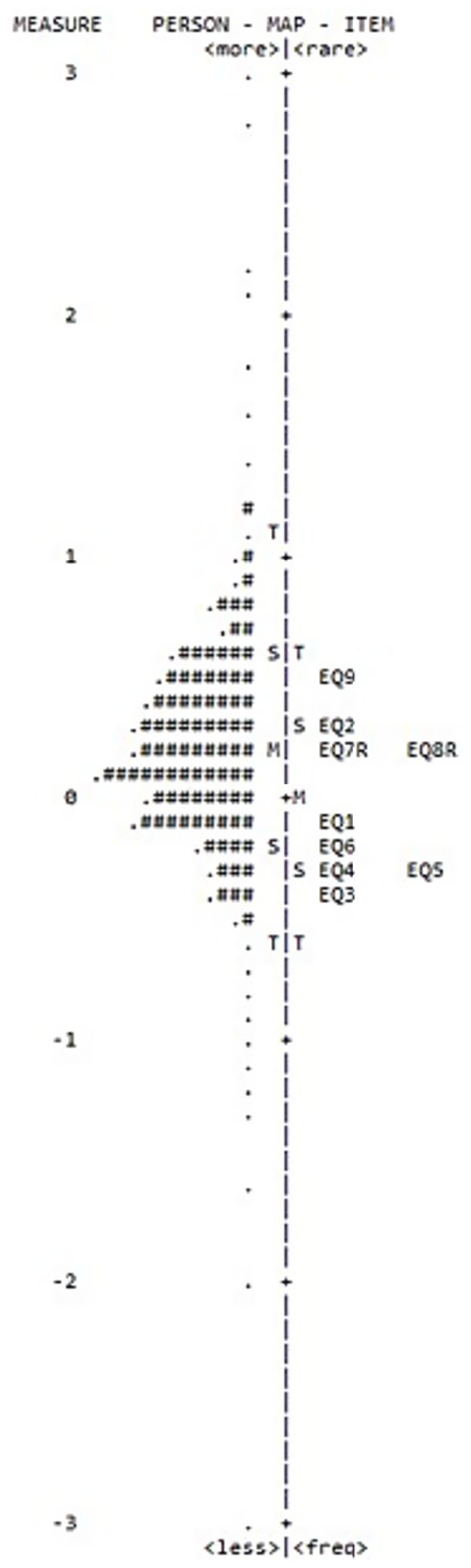
Person—item map.

In Figure 2 both the location of person abilities and item difficulties, respectively, along the same latent dimension were plotted on the same logit scale. The top part of the figure depicted the most able people and the most difficult items (EQ9), while the less able people and the less difficult items (EQ3) were positioned in the bottom part of the figure.

The item-person map shows the effectiveness of the overall targeting of the scale with no gaps in the empirical item hierarchy: the item locations ranged from −0.39 (EQ3) to +0.50 (EQ9) logits, implying that the EQ items were well spread over the logits scale. Although the item distribution adequately covers the central portion of the latent continuum for a brief 9-item scale, the overall range remains modest, suggesting that individuals with very high or very low levels of existential quest may be less precisely represented.

The dispersion of the item calibrations was statistically determined by calculating item strata, which was 2.8 (PSI = 1.85) for this scale of nine items. This finding confirmed that the difficulty of the items defining the empirical item hierarchy has sufficient variance and the scale was able to distinguish approximately three distinct levels of person existential beliefs and thoughts.

**Figure 3:**
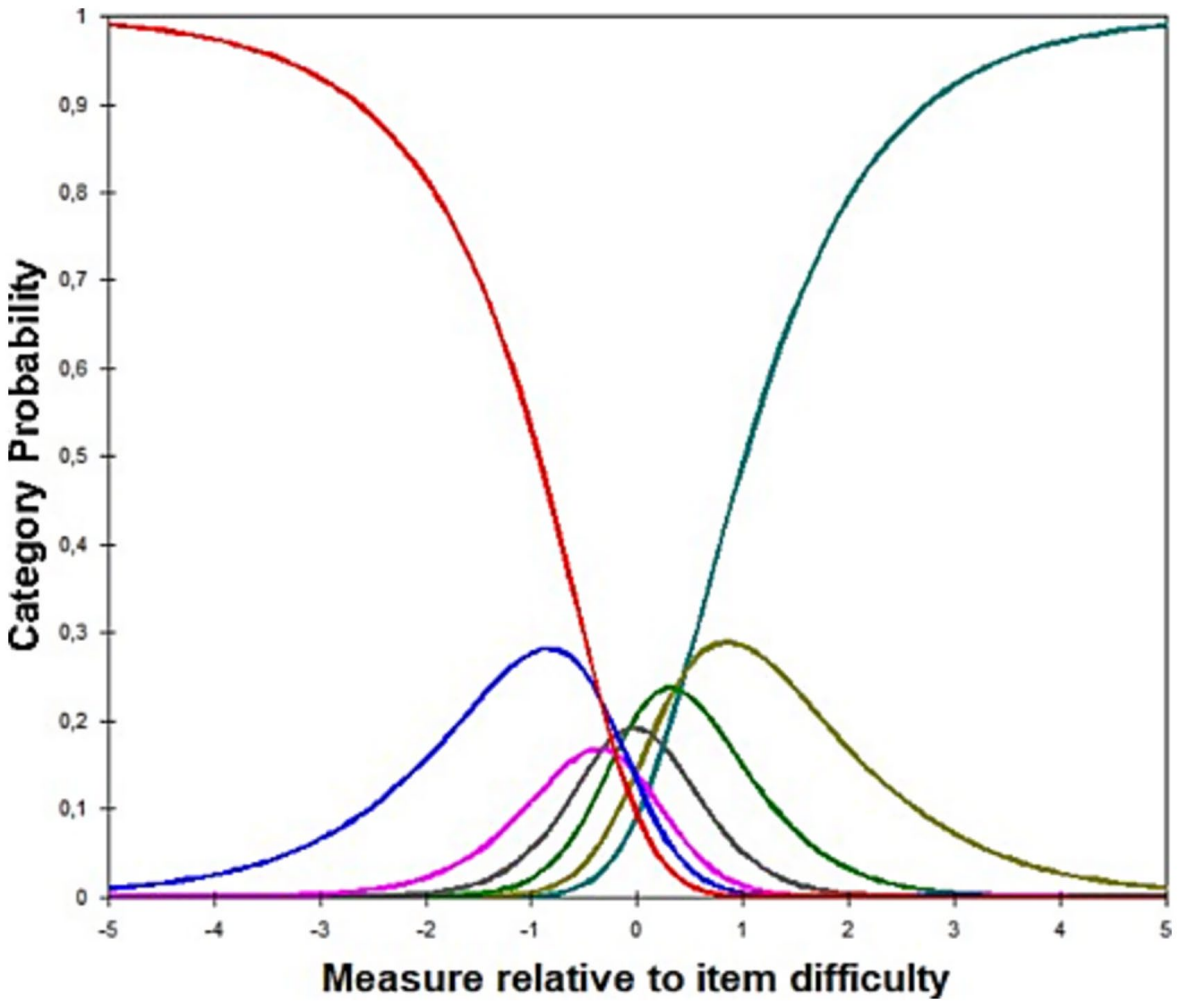
ICC.

Examining category thresholds enables assessment of whether respondents used the rating scale in an ordered and interpretable manner, with higher categories reflecting progressively higher levels of the latent trait. In our data, thresholds were formally ordered (*τ*₁ = −1.88, τ₂ = −0.83, τ₃ = −0.36, τ₄ = −0.03, τ₅ = 0.32, τ₆ = 0.85, τ₇ = 1.99); however, inspection of the ICC indicated that some intermediate categories did not exhibit clearly differentiated probability peaks (Figure 3). This suggests limited semantic distinction among adjacent response options, despite the formal ordering of thresholds, revealing a pattern commonly observed in rating scales with many response categories, where respondents may not reliably discriminate among certain intermediate levels.

To further examine these issues, we re-estimated the model after removing the reversed item (EQ7) and collapsing the seven response categories into five. While collapsing the categories improved threshold coherence, it did not substantially alter the global model fit in the nine-item structure, as both reversed items continued to display the highest misfit. Removing EQ7 improved dimensionality and item fit to some extent; however, this solution left EQ8R as the sole reversed item. This configuration was less defensible from a measurement perspective and is not typically recommended, as isolated reversed items tend to introduce construct-irrelevant variance. For this reason, the eight-item model was not considered a viable final version of the scale, despite its modest empirical gains (see Supplementary material for full comparative analyses). In line with this rationale, all uniform differential item functioning (DIF) analyses across sex, age group and religion were conducted on the theoretically coherent nine-item, seven-response category version of the scale. The invariance of the instrument was confirmed for both sex and age, as well as for whether or not one belongs to a religion: all DIF contrasts were <0.43 and thus negligible, as shown in Table 4.

**Table 4:** DIF analysis.

DIF contrast*			
Item	Sex	Age	Religion
	Male vs. female	18–31 vs. older than 31	Not religion believer vs. religion believer
EQ1	0.00	−0.13	−0.07
EQ2	0.15	−0.13	0.25
EQ3	−0.12	0.08	−0.24
EQ4	−0.08	0.04	−0.19
EQ5	0.04	−0.12	−0.04
EQ6	0.03	0.00	0.04
EQ7(R)	−0.02	0.00	0.00
EQ8(R)	−0.14	0.30	−0.02
EQ9	0.11	0.00	0.20

^*^DIF contrast ranges: <0.43 = negligible; (0.43; 0.64) = moderate; >0.64 = large.

## 4 Discussion

The present study extends previous CTT-based validations of the EQS by providing item-level evidence through Rasch modeling. While previous work has established acceptable internal consistency and factor structure, the current analysis offers stronger support for construct validity by examining item adaptation, response category functioning, scale targeting, and measurement invariance. These properties could not be assessed using traditional CTT approaches and therefore represent a substantial advance in understanding the psychometric performance of the EQS across different respondents. Overall, the scale demonstrated excellent psychometric performance within the Rasch framework: all items showed acceptable fit statistics, the scale was appropriately targeted to the sample, and no significant differences emerged by gender, age, or religious affiliation. These results indicate that the EQS captures a coherent latent construct and operates consistently across different subgroups. Although existential quest is conceptually multifaceted—including uncertainty about existential questions, openness to reconsidering one’s perspective, and the valorization of doubt—Rasch analysis supported a single underlying latent dimension. This theoretical-empirical tension is not unexpected for a brief instrument designed to capture an overarching disposition rather than discrete subcomponents. A unidimensional structure is therefore theoretically coherent: the items appear to function as different expressions of the same general tendency toward flexible engagement with existential questions. In this sense, the finding of unidimensionality aligns with the conceptualization of EQ as a broad attitudinal orientation.

However, some specifics need to be considered to improve the validity of EQS and its usefulness in social and human science research.

First, Rasch analyses confirmed the non-optimal functioning of one item (EQ7: I know perfectly well what the goal of my life is). This reversed-item seems to be redundant compared to the first item (EQ1: Today, I still wonder about the meaning and goal of my life) because it still measures the same problem of uncertainty in relation to existential quest. Although reverse-worded items are often retained in questionnaires to mitigate acquiescence bias, the present findings suggest that EQ7 may not fulfill this methodological function effectively. Its consistently lower PT-measure correlation, combined with recurrent misfit reported in previous studies, indicates that the item may introduce construct-irrelevant variance rather than improving response quality. Thus, while the inclusion of a reverse-coded item can be valuable in principle, in the case of the EQS this specific item appears to weaken the scale’s internal coherence. This suboptimal functioning is consistent with previous studies reporting similar issues (Van Pachterbeke et al., 2012; Miglietta et al., 2023; Rizzo et al., 2022). Consequently, it may be advisable to revise or remove the item from the scale (Rizzo et al., 2019). Any modification should, however, be grounded in further empirical evaluation, ideally through experimental testing of alternative reverse-coded formulations that preserve the construct’s conceptual clarity.

Second, results on the inspection of logit values showing person abilities and item difficulties reported a good variance of the entire scale. Specifically, people were well distributed along the scale and no ceiling or floor effects were found. However, the modest spread of item difficulties indicates that, while the scale is suitably targeted to most respondents, extreme levels of existential quest may require additional items to achieve optimal precision. In addition, the contrast between the most difficult item (EQ9: “I often re-evaluate my opinion on religious/spiritual beliefs”) and the easiest (EQ3: “Being able to doubt one’s beliefs and re-evaluate them is a good quality”) can be understood more appropriately from a psychometric point of view. The greater difficulty of EQ9 suggests that re-evaluating religious or spiritual beliefs is a form of reflection that is less accessible cognitively or less frequently activated for many respondents, particularly when such beliefs are not relevant to their everyday thinking. In contrast, EQ3 draws on a broad and socially familiar metacognitive position, namely the positive evaluation of doubt, which is generally easier for respondents to endorse. These differences in item positioning likely reflect variation in the cognitive accessibility and generality of the underlying reflective processes rather than differences in respondents’ personal life experiences. Although social desirability may contribute to the high approval rates observed for EQ3, given that rejecting doubt may be perceived as socially undesirable, this remains a speculative interpretation and is not corroborated by the current data. Further research incorporating direct measures of social desirability or using experimental designs would be needed to examine this possibility.

Third, regarding the inspection of ICC, it is evident that participants did not use all the seven categories in response to the items. Specifically, they tend to select the extreme categories (1 = completely disagree; 7 = completely agree). These results suggest that participants may experience difficulty in providing a clear or fair self-assessment of their flexibility or inflexibility regarding existential issues. It tends to be easier to have a clear opinion on this sensitive topic than to recognise a nuance in the level of agreement/disagreement. Although from a methodological perspective it would be desirable to have four response categories, it may be prudent to reduce the number of categories from seven to five in order to retain the neutral or undecided option, which is important for understanding those who do not have a well-defined idea of a complex construct such as existential quest.

Finally, our results demonstrated robust measurement invariance of the EQS with regard to sex, age and religion: DIF analyses showed that men and women, young adults and adults, and those who describe themselves as religious perform equally to those who are not. The evidence of measurement invariance across gender, age, and religious affiliation represents a substantial strength of the EQS. These results indicate that the scale captures existential flexibility comparably across demographic and ideological groups, consistent with the original goal of developing a measure unrelated to specific religious orientations (Van Pachterbeke et al., 2012). This level of invariance also strengthens the EQS’s suitability for comparative research, as it supports the interpretation that observed score differences reflect genuine variations in existential seeking rather than measurement bias. Future studies could explore this potential further by examining invariance across additional comparison groups and contexts.

An additional limitation concerns the contextual conditions under which the EQS was administered. The datasets included in this study did not assess situational stress, exposure to critical life events, or emotionally demanding contexts. As a result, the present findings cannot speak to how the EQS performs under conditions of heightened existential tension. Future research may therefore examine the scale in samples more frequently confronted with stressful or tragic experiences—such as healthcare workers or funeral professionals—to determine whether existential questioning operates similarly across contexts.

Furthermore, the association between EQ and open-mindedness (Saroglou, 2025) can be more explicitly understood within the broader psychological framework underlying existential reflection. Engaging with existential quest inherently involves a willingness to reconsider one’s assumptions and tolerate the uncertainty that arises when one’s beliefs are questioned. This connection situates EQ within a constellation of metacognitive and epistemic dispositions rather than presenting it as an isolated trait. Clarifying this theoretical grounding also strengthens the rationale for future research on the potential implications of EQ for wellbeing, acknowledging that both beneficial and challenging consequences of existential quest may coexist.

## 5 Conclusion

The EQ scale has demonstrated robust validity and reliability across extensive datasets, offering valuable insights for researchers in the social and human sciences. As a validate measure, it effectively encompasses both religious and non-religious respondents, making it applicable to diverse studies within the social sciences. Moreover, despite its short format of just nine items, the instrument adeptly assesses the flexibility inherent in existential quests. This brevity is particularly advantageous in social science and human research, where the integration of multiple constructs is often essential for comprehensive analysis.

## Data Availability

The data analyzed in this study is subject to the following licenses/restrictions: We did not obtain written consent from the participants for the publication of the dataset. Requests to access these datasets should be directed to giorgia.molinengo@unito.it.
