# Supplementary material for: Validating the Existential Quest Scale using item response theory
Source: Front Psychol. 2026 Jan 9;16:1716603. doi: 10.3389/fpsyg.2025.1716603 (PMC12827577; doi:10.3389/fpsyg.2025.1716603)
Supplement: Supplementary file 1 [file Supplementary_material.docx]

**Supplementary material**

The following tables report the comparison of results obtained from the Partial Credit Model estimations of the full **9-item Existential Quest Scale (EQS)** and the reduced **8-item version** after removal of item EQ7.

Table A1. Comparison of Global Rasch Fit Indices Between the 9-Item Model and the 8-Item Model (EQ7 Removed)

| Index | 9-Item Model | 8-Item Model | Interpretation |
| --- | --- | --- | --- |
| Variance explained by Rasch measures | 36.3% | 41.4% | Higher explained variance indicates improved measurement coherence after removal of EQ7. |
| Unexplained variance (total) | 63.7% | 58.6% | Reduction reflects lower residual noise and improved model–data correspondence. |
| First residual contrast (eigenvalue) | 1.89 | 1.72 | Slight decrease suggests weaker secondary dimensions. |
| First residual contrast (% of unexplained variance) | 13.4% | 12.6% | Marginal decrease consistent with improved unidimensionality. |
| Item reliability | 0.97 | 0.98 | Both values indicate high item stability; the change is negligible. |

Table A2. Item Statistics for the 8-Item Model (without EQ7)

| ITEM | LOCATION  Logits | INFIT  MNSQ | OUTFIT  MNSQ | PT-Measure  correlation |
| --- | --- | --- | --- | --- |
| EQ8(R) | .26 | 1.66 | 1.70 | .32 |
| EQ1 | –.05 | 1.13 | 1.16 | .52 |
| EQ9 | .56 | 1.02 | 1.05 | .55 |
| EQ2 | .35 | .99 | .99 | .59 |
| EQ6 | –.16 | .89 | .93 | .50 |
| EQ3 | –.39 | .79 | .77 | .58 |
| EQ5 | –.26 | .74 | .74 | .61 |
| EQ4 | –.31 | .73 | .73 | .60 |

Table A3. Comparison of Item Misfit between the 9-Item Model and the 8-Item Model (EQ7 Removed)

| Item | Infit  (9-item) | Infit  (8-item) | Change | Outfit  (9-item) | Outfit  (8-item) | Change |
| --- | --- | --- | --- | --- | --- | --- |
| EQ8R | 1.44 | 1.66 | Slight deterioration | 1.54 | 1.70 | Deterioration |
| EQ7R | 1.33 | — | Removed | 1.44 | — | Removed |
| EQ1 | 1.09 | 1.13 | Minimal increase | 1.08 | 1.16 | Minimal increase |
| EQ9 | 1.01 | 1.02 | Stable | 1.02 | 1.05 | Stable |
| EQ2 | 1.00 | 0.99 | Stable | 0.99 | 0.99 | Stable |
| EQ6 | 0.85 | 0.89 | Small increase | 0.88 | 0.93 | Small increase |
| EQ3 | 0.78 | 0.79 | Stable | 0.75 | 0.77 | Stable |
| EQ5 | 0.76 | 0.74 | Minimal decrease | 0.74 | 0.74 | Stable |
| EQ4 | 0.73 | 0.73 | Stable | 0.72 | 0.73 | Stable |

The following results illustrate the additional analysis performed to evaluate whether reducing the response scale from **7 categories** to **5 categories** improves measurement functioning while retaining all **9 original EQS items.**

Table A4. Global Fit Indices for the 9-Item Models using 7 category vs. 5 categories of the rating scale

| Index | Model A: 9 items, 7 categories | Model B: 9 items, 5 categories | Interpretation |
| --- | --- | --- | --- |
| Variance explained by Rasch measures | 36.3% | 35.5% | Comparable explained variance; category collapsing does not improve model fit. |
| Unexplained variance (1st contrast) | 1.89 (13.4%) | 1.86 (13.3%) | Dimensionality essentially unchanged. |
| Essential unidimensionality | ~55–56% | 50.8% | Decrease indicates a weaker primary dimension after collapsing categories. |
| Item reliability | 0.97 | 0.98 | High in both models; change negligible. |

Table A5. Item Fit Statistics (Infit/Outfit) for the Two 9-Item Models

| Item | 7-category INFIT | 5-category INFIT | 7-category OUTFIT | 5-category OUTFIT |
| --- | --- | --- | --- | --- |
| EQ8R | 1.44 | 1.40 | 1.54 | 1.52 |
| EQ7R | 1.33 | 1.31 | 1.44 | 1.42 |
| EQ1 | 1.09 | 1.09 | 1.08 | 1.08 |
| EQ9 | 1.01 | 1.02 | 1.02 | 1.02 |
| EQ2 | 1.00 | 0.99 | .99 | .98 |
| EQ6 | .85 | .86 | .88 | .88 |
| EQ3 | .78 | .79 | .75 | .77 |
| EQ5 | .76 | .76 | .74 | .76 |
| EQ4 | .73 | .74 | .72 | .73 |

Consistent with the findings reported in the main manuscript, reducing the rating scale from seven to five response categories does not produce a substantial improvement in global model fit or dimensionality. In contrast, removing the EQ7 item produces measurable gains in explained variance and a reduction in residual contrasts; however, this modification results in a scale containing a single reverse-coded item, a psychometrically unusual and potentially problematic configuration.

It is important to emphasize that all additional analyses presented here were conducted on the same dataset used for the primary Rasch calibration. Therefore, they should be interpreted as preliminary and exploratory: post hoc recoding and item removal cannot predict how respondents would perform when presented with a shortened scale or an item set containing only a reversed item. Future validation studies using revised item sets and response formats are needed before recommending any structural modifications to the EQS.
